# Postoperative Outcomes of Minimally Invasive Versus Conventional Off-Pump Coronary Artery Bypass Within an ERACS Protocol: A Matched Analysis

**DOI:** 10.3390/jcm15010328

**Published:** 2026-01-01

**Authors:** Mostafa Saad, Ibrahim Gadelkarim, Michael Borger, Massimiliano Meineri, Aniruddha Janai, Sophia Sgouropoulou, Jörg Ender, Waseem Zakhary

**Affiliations:** 1 Department of Anaesthesia and Intensive Care Medicine, Leipzig Heart Center, 04289 Leipzig, Germany; mostafa.saad@helios-gesundheit.de (M.S.); massimiliano.meineri@helios-gesundheit.de (M.M.); aniruddha.janai@helios-gesundheit.de (A.J.); sophia.sgouropoulou@helios-gesundheit.de (S.S.); joerg.ender@helios-gesundheit.de (J.E.); 2 Department of Cardiac Surgery, Leipzig Heart Center, 04289 Leipzig, Germany; ibrahim.gadelkarim@helios-gesundheit.de (I.G.); michael.borger@helios-gesundheit.de (M.B.)

**Keywords:** MICS-CABG, OPCAB, ERACS, early extubation, minimally invasive cardiac surgery, postoperative recovery

## Abstract

**Background/Objectives**: Minimally invasive coronary artery bypass grafting (MICS-CABG) offers reduced access trauma compared with conventional off-pump coronary artery bypass (OPCAB) but requires more demanding surgical and anesthetic conditions, including single-lung ventilation. Enhanced Recovery After Cardiac Surgery (ERACS) pathways—particularly those incorporating early extubation in a post-anesthesia care unit (PACU) and routine ICU bypass—may harmonize postoperative recovery across different surgical approaches. This study evaluated whether a standardized early-extubation ERACS protocol could achieve comparable short-term recovery outcomes between MICS-CABG and OPCAB. **Methods**: This single-center retrospective study included all adult patients who underwent off-pump MICS-CABG via mini-thoracotomy or OPCAB via sternotomy between January 2020 and December 2024 within an ERACS pathway. Propensity score matching (1:1) was applied using key demographic and clinical variables. Primary outcomes were hospital length of stay (LOS), ventilation time, and unplanned ICU transfer. Secondary outcomes included postoperative complications, transfusion requirements, pain scores, and in-hospital mortality. **Results**: Of 144 MICS-CABG patients, 131 met inclusion criteria and 116 were propensity-matched to 116 OPCAB patients. Operative duration was longer in MICS-CABG (238.9 ± 65 vs. 175.0 ± 48 min; *p* < 0.001). However, ventilation time (112.2 ± 56.9 vs. 116.9 ± 64.7 min; *p* = 0.59), hospital LOS (8.7 ± 4.0 vs. 8.6 ± 4.1 days; *p* = 0.78), and unplanned ICU transfer (0.9% vs. 2.6%; *p* = 0.37) were comparable. Postoperative complications, transfusion rates, pain scores, and in-hospital mortality also did not differ significantly. **Conclusions**: Within a structured ERACS pathway incorporating early extubation and ICU bypass, MICS-CABG and OPCAB achieved similar short-term recovery outcomes despite differences in operative complexity. These findings suggest that ERACS can provide a consistent postoperative recovery framework across both revascularization strategies.

## 1. Introduction

Minimally invasive surgery emerged in general surgery in the late 1980s, when the first video-assisted cholecystectomies demonstrated that smaller incisions could shorten recovery without compromising safety [1]. Cardiac surgeons adopted minimally invasive approaches more cautiously, given the need for reliable myocardial protection and full coronary exposure. In 2009, McGinn and colleagues described minimally invasive coronary artery bypass grafting (MICS-CABG)—a multivessel, hand-sewn revascularization performed on the beating heart through a 4–6 cm left anterior mini-thoracotomy. Their dual-center series of 450 consecutive patients demonstrated complete revascularization without conversion in 96.2%, a conversion rate of 3.8%, and a hospital mortality of 1.3% [2]. Subsequent studies have consistently confirmed the feasibility and safety of this technique [3,4,5,6,7].

Parallel to these surgical advancements, peri-operative teams have increasingly adopted Fast-track pathway [8] and Enhanced Recovery after Surgery (ERAS) principles. In cardiac surgery, the 2019 ERAS Cardiac Society update by Engelman et al. [9] and the 2024 joint consensus statement [10] codified more than 30 evidence-based elements—from prehabilitation and lung-protective ventilation to opioid-sparing multimodal analgesia and early mobilization—aimed at attenuating surgical stress and accelerating recovery.

Although both MICS-CABG and off-pump coronary artery bypass grafting (OPCAB) avoid cardiopulmonary bypass–related complications, the two procedures differ substantially in surgical access and physiologic demands. MICS-CABG requires a mini-thoracotomy, single-lung ventilation (SLV), and is generally more complex with longer operative times, whereas OPCAB is performed through a full sternotomy with shorter access and exposure times.

This study aimed to evaluate the impact of a standardized ERACS protocol on postoperative recovery outcomes in patients undergoing either minimally invasive or conventional off-pump coronary revascularization.

We hypothesized that, despite the surgically and anesthesiologically demanding nature of MICS-CABG, the ERACS protocol would be equally feasible compared with OPCAB and would result in comparable postoperative recovery outcomes. Primary endpoints were hospital length of stay, ventilation time, and rates of direct ICU transfer. Secondary endpoints included postoperative complications, transfusion requirements, pain scores, and in-hospital mortality.

## 2. Materials and Methods

### 2.1. Ethical Statement

This retrospective study was approved by the local ethics committee with the approval number (305/25-ek) and conducted at a single center (Heart Center Leipzig). The requirement for individual informed consent was waived due to the retrospective design.

### 2.2. Patient Population

We included all patients who underwent minimally invasive coronary artery bypass grafting on a beating heart via lateral thoracotomy (MICS CABG), without the use of cardiopulmonary bypass (CPB), and were managed under an Enhanced Recovery After Cardiac Surgery (ERACS) protocol between 1 January 2020 and 31 December 2024. These patients were compared with patients who underwent off-pump coronary artery bypass grafting via median sternotomy (OPCAB) in the same time frame. Exclusion criteria were age < 18 years, use of CPB, direct postoperative ICU admission, and intraoperative conversion to sternotomy.

### 2.3. Endpoints

**Figure 1:**
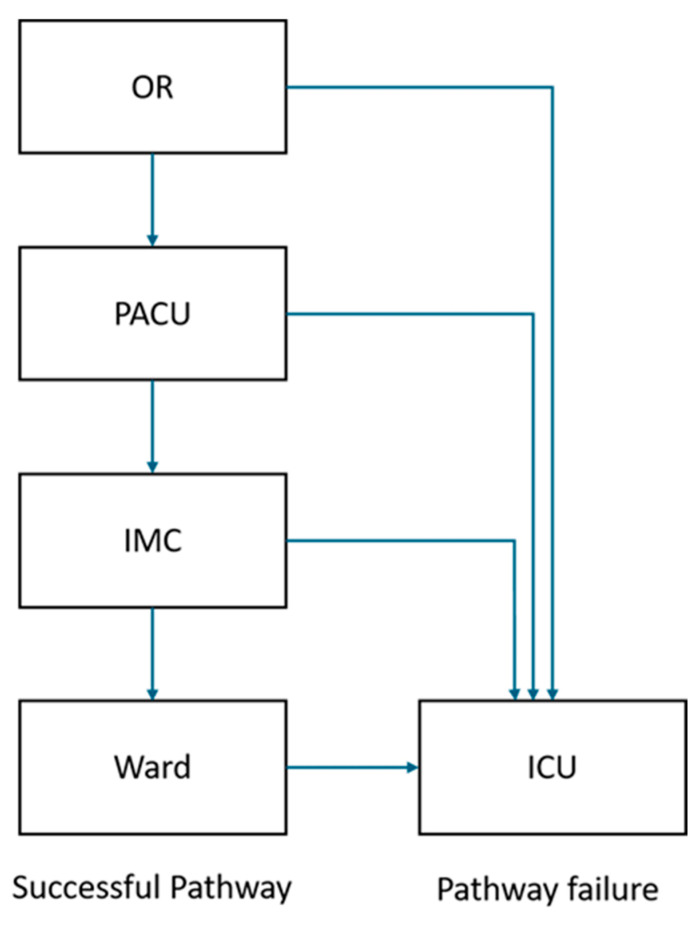
Patient pathway.

Primary outcomes were total hospital length of stay (LOS), duration of mechanical ventilation (defined as time from arrival to the PACU to extubation), and the rate of unplanned ICU transfer. According to institutional workflow, patients were ideally transferred from the operating room to the PACU, then to the intermediate care unit (IMC) on the same day, and subsequently to the regular ward the following day (Figure 1). The PACU operated Monday to Friday from 10:00 am to 10:30 pm, with the last permissible admission at 06:00 pm. Patients finishing surgery after this time were transferred directly to the ICU.

Secondary endpoints included:

Postoperative pain intensity, assessed at regular intervals using the Numeric Rating Scale (NRS, 0–10), with a target score < 4.

Postoperative myocardial infarction, diagnosed based on typical ECG changes, elevated cardiac biomarkers (CK-MB), and compatible clinical presentation.

Postoperative pulmonary complications, defined as newly developed pulmonary infiltrates on chest radiographs in combination with clinical signs of respiratory infection or impaired gas exchange.

Acute renal failure, defined as a postoperative increase in serum creatinine ≥ 1.5 times the preoperative baseline.

Postoperative delirium, assessed using the Intensive Care Delirium Screening Checklist (ICDSC) in the PACU and IMC, and the Nursing Delirium Screening Scale (Nu-DESC) on the ward; a score ≥ 2 on either scale was considered positive.

Significant postoperative bleeding, defined as total chest tube output > 400 mL within the first 24 h.

In-hospital mortality, defined as death from any cause occurring during the index hospitalization.

### 2.4. Anesthetic Management

No routine premedication was administered the night before surgery. Patients fasted for 6 h but were allowed unrestricted clear fluids preoperatively. Before induction, an invasive arterial line was placed. Induction consisted of propofol (1–2 mg/kg IV), sufentanil (0.3–0.7 μg/kg IV) or fentanyl (1–2 μg/kg IV), and neuromuscular blockade with either atracurium (0.3–0.6 mg/kg IV) or rocuronium (0.3–0.6 mg/kg IV).

Patients in the MICS-CABG group were intubated with a double-lumen tube (DLT) (Ruesch^®^, Teleflex Medical, Wayne, PA, USA), with correct positioning confirmed by bronchoscopy. Complete deflation of the left lung was required for optimal exposure, and single-lung ventilation (SLV) was initiated before thoracotomy and maintained throughout the coronary grafting stage. Patients in the OPCAB group were intubated with a single-lumen tube. A triple-lumen central venous catheter and an 8.5 F introducer sheath were inserted in all patients.

Anesthesia was maintained with either propofol infusion (3–6 mg/kg/h IV) or sevoflurane (minimum alveolar concentration 0.8–1.1), supplemented by a sufentanil infusion at 1 μg/kg/h before incision and 0.25–0.5 μg/kg/h after thoracotomy or sternotomy.

Packed red blood cells were transfused when the hematocrit fell below 25%.

Core temperature was monitored using a nasopharyngeal probe and a bladder thermistor via urinary catheter. Forced-air warming (3M™ Bair Hugger™, St. Paul, MN, USA) was initiated before induction and continued until PACU transfer to maintain a core temperature ≥ 36 °C; it was continued in the PACU if temperature remained < 36 °C. All infusions and blood products were warmed using the LEVEL 1 HOTLINE^®^ Blood and Fluid Warmer (ICU Medical, San Clemente, CA, USA).

### 2.5. Surgical Approach Selection and Operative Technique

At our institution, the choice between minimally invasive coronary artery bypass grafting (MICS-CABG) via left mini-thoracotomy and conventional off-pump coronary artery bypass grafting via median sternotomy (OPCAB) is guided by a predefined, patient-centered selection strategy, rather than by surgical access alone. This strategy integrates coronary anatomy, physiological reserve, comorbidities, technical feasibility, and anticipated recovery goals, in line with contemporary guideline recommendations and institutional expertise [11,12].

Patient selection strategy

The main eligibility criteria for MICS-CABG were: (1) surgeon proficiency in the technique, (2) adequate lung function (forced expiratory volume in 1 s > 50% to 80% of the predicted value or PaO_2_ > 60 mm Hg and PaCO_2_ < 55 mm Hg on room air), and (3) adequate distal coronary runoff without heavy calcification or intramuscular coronary courses that could limit exposure. Exclusion criteria included: (1) expected intrathoracic adhesions due to previous cardiac surgery or prior radiation therapy, (2) severe forms of pectus excavatum or other chest wall deformity, (3) inability to tolerate single-lung ventilation and (4) acute myocardial infarction in the past 30 days.

Conversely, OPCAB via median sternotomy is preferentially selected for patients with multivessel coronary artery disease requiring more extensive revascularization, complex or diffusely diseased coronary anatomy, or in the presence of significant comorbidities such as advanced age, chronic lung disease, renal dysfunction, diabetes mellitus, left ventricular dysfunction, or prior cardiac surgery. OPCAB is also favored when severe ascending aortic calcification is present, allowing avoidance of aortic manipulation while maintaining procedural flexibility.

As a consequence of this intentional selection process, the two surgical cohorts represent distinct but clinically appropriate therapeutic populations, reflecting real-world decision-making rather than random treatment allocation.

OPCAB group

Patients in the OPCAB group underwent coronary revascularization via full median sternotomy. Graft configuration followed our institutional strategy for single-, multiple-, or total-arterial revascularization. Single-arterial revascularization supplemented by venous grafts was primarily used in elderly patients (>72 years) or in those with poor distal coronary targets. The radial artery was employed in cases of high-grade coronary stenosis with favorable distal runoff. Bilateral internal mammary artery (BIMA) grafting was reserved for patients without type 1 diabetes mellitus or morbid obesity due to the increased risk of sternal wound complications.

Myocardial stabilization was achieved using the Octopus^®^ tissue stabilizer (Medtronic, Minneapolis, MN, USA), with routine use of intracoronary shunts. Anastomoses were performed using standard techniques with 7-0 or 8-0 Prolene sutures. Graft patency and flow were assessed after each anastomosis using transit-time flow measurement, targeting a mean flow > 15 mL/min and a pulsatility index < 5 [13].

MICS-CABG group

MICS-CABG was performed through a left anterolateral mini-thoracotomy under single-lung ventilation, allowing direct-vision harvesting of the left (and when indicated right) internal mammary artery and direct-vision coronary anastomosis. Stabilization techniques, intracoronary shunt use, anastomotic methods, and transit-time flow assessment were identical to those employed in the OPCAB group.

In contrast to OPCAB, graft selection in the MICS-CABG group was more liberal with respect to BIMA use, as the absence of median sternotomy eliminates the risk of sternal wound complications. Obesity alone was not considered a contraindication for the minimally invasive approach in otherwise suitable patients.

### 2.6. PACU Management

At the end of surgery, if PACU admission criteria were met and agreed upon by both the surgeon and anesthesiologist, patients were transported intubated to the PACU. Admission criteria included hemodynamic stability with or without low-dose vasopressor and/or inotropic support (norepinephrine ≤ 0.1 μg/kg/min and/or epinephrine ≤ 0.05 μg/kg/min) and a core temperature ≥ 36 °C [14].

Upon arrival, all female patients received droperidol 1.25 mg IV; ondansetron 4 mg IV was added for patients with a history of postoperative nausea and vomiting [15].

For postoperative analgesia, patients received metamizole 1 g IV and piritramide 0.1 mg/kg IV. Additional piritramide boluses (0.02–0.03 mg/kg IV) were administered as needed to maintain NRS < 4.

Patients were extubated when all criteria listed in Table 1 were fulfilled.

After extubation, patients were placed in a semi-sitting position. Non-invasive ventilation (NIV) was applied for at least 30 min and repeated as required based on arterial blood gas analysis. Patients were encouraged to use a Triflow^®^ incentive spirometer (Teleflex, Morrisville, NC, USA) to improve gas exchange and reduce atelectasis. Clear fluid intake was encouraged once adequate swallowing was confirmed.

Patients remained in the PACU until criteria for step-down were met. They were then transferred to the intermediate care unit (IMC) according to Table 2.

### 2.7. ERACS Program

The local ERACS protocol is comprehensive and incorporates most class I and II recommendations mentioned in previous guidelines [9,10]. Key intraoperative components include routine administration of tranexamic acid, unrestricted fluid intake before anesthesia, perioperative glycemic control following the Portland protocol, antibiotic-based surgical-site–infection prophylaxis, lung-protective ventilation and active temperature management to avoid hypothermia. Transesophageal echocardiography and goal-directed hemodynamic therapy were used routinely. A multimodal, opioid-sparing analgesic strategy is applied in all patients.

Postoperatively, all patients undergo structured delirium screening using ICDSC (in PACU and IMC) and Nu-DESC (on the ward). Early mobilization followed the ICU liberation bundle, and an early extubation strategy targeted extubation within two hours in the PACU, aiming to bypass ICU admission entirely.

Chest tube patency was maintained via continuous −20 cmH_2_O suction, whereas routine tube stripping was avoided. Additional standardized measures include thromboprophylaxis, PONV prophylaxis [15], comprehensive blood management using predefined transfusion thresholds and viscoelastic testing (Quantra^®^, HemoSonics, LLC, Durham, NC, USA).

The program emphasized multidisciplinary team involvement and continuous quality improvement through routine auditing to ensure adherence and process optimization.

### 2.8. Data Collection

The primary anesthesiologist scanned the anesthesia record and PACU observation chart using Medlinq^®^ version 4.2.1 (Medlinq Softwaresysteme GmbH, Hamburg, Germany). Manual corrections were permitted prior to finalization in case of illegible handwriting or inconsistencies. Data were then collected retrospectively from the clinical information systems iMedOne^®^ (Deutsche Telekom Healthcare and Security Solutions GmbH, Bonn, Germany), Clemens^®^ (Teratec GmbH, Münster, Germany), and the Medlinq^®^ patient chart.

### 2.9. Statistical Analysis

Given that the choice between MICS-CABG and OPCAB at our institution is based on predefined clinical, anatomical, and physiological selection criteria rather than random allocation, baseline differences between treatment groups were anticipated. To account for these intentional selection-related differences and to facilitate comparison of early postoperative recovery outcomes within a shared ERACS pathway, propensity score matching (PSM) was performed.

The propensity score was estimated using a logistic regression model incorporating clinically relevant preoperative and intraoperative variables known to influence both surgical approach selection and postoperative outcomes, including age, sex, body mass index, left ventricular ejection fraction, history of myocardial infarction, redo surgery, surgical urgency, EuroScore II, and number of grafts. While PSM was used to reduce measurable baseline imbalances between groups, it cannot account for unmeasured confounders inherent to retrospective observational studies; therefore, comparisons should be interpreted as adjusted associations rather than causal effects.

MICS CABG patients were matched 1:1 without replacement to OPCAB patients using nearest-neighbor matching with a caliper width of 0.2 of the standard deviation of the logit of the propensity score. Balance after matching was assessed using standardized mean differences, with an absolute SMD < 0.1 considered negligible imbalance. Post-matching, perioperative and postoperative outcomes were compared between the two groups.

Normality of continuous variables was assessed using the Shapiro–Wilk test. Between-group Normality of continuous variables was assessed using the Shapiro–Wilk test. Between-group comparisons were conducted with the Mann–Whitney U-test for continuous variables and the Chi-square or Fisher’s exact test for categorical variables, as appropriate. Continuous variables are presented as mean (standard deviation, SD) and median [interquartile range, IQR], and categorical variables as absolute numbers and percentages. Effect sizes are reported as mean differences (MDs) for continuous variables and odds ratios (ORs) with 95% confidence intervals (CIs) for categorical variables.

All statistical tests were two-sided, with *p*-values < 0.05 considered statistically significant. Analyses were performed using SPSS Statistics 25.0 (IBM, Chicago, IL, USA) and StatsDirect 3.0 (StatsDirect Ltd., Cheshire, UK).

## 3. Results

**Figure 2:**
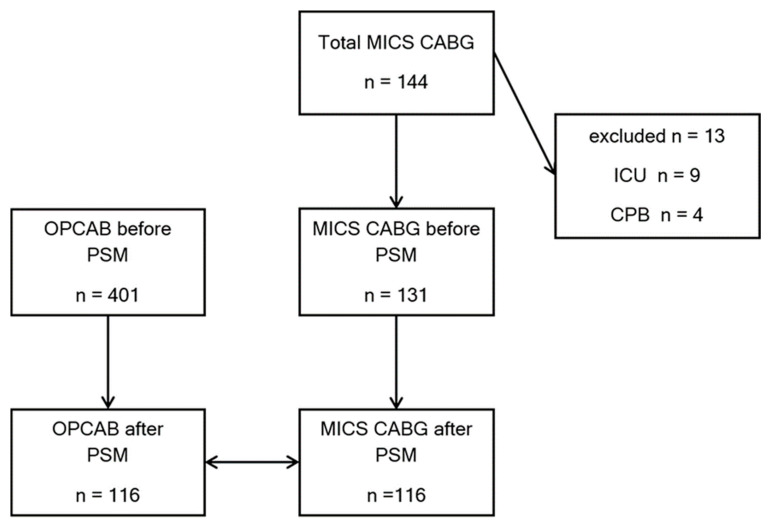
Patients Flow-chart. PSM: Propensity score match.

Between January 2020 and December 2024, a total of 144 patients underwent MICS-CABG at the Heart Center Leipzig. Of these, 9 patients were excluded due to direct ICU transfer (because the last permissible PACU admission time was exceeded) and 4 patients were excluded due to planned or unplanned conversion to cardiopulmonary bypass (CPB) because single-lung ventilation was not tolerated. The remaining 131 MICS-CABG patients were included in the propensity score analysis and matched to patients who underwent OPCAB during the same period. Propensity score matching yielded two well-balanced groups of 116 patients each (Figure 2).

**Figure 3:**
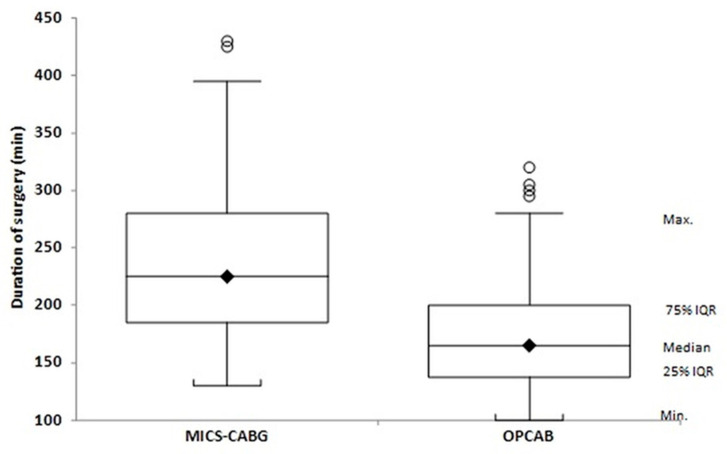
Duration of surgery.

After PSM, demographic and operative characteristics were comparable between the two groups (Table 3), with the exception of operative duration, which was significantly longer in the MICS-CABG group (238.9 ± 65 vs. 175.0 ± 48 min; *p* < 0.001) (Figure 3).

Ventilation time, PACU length of stay, and hospital length of stay were comparable between groups (Table 4). ICU transfer was rare in both cohorts.

Overall postoperative complication rates were comparable in both groups (Table 5).

## 4. Discussion

This study demonstrated that although operative duration was significantly longer in the MICS-CABG group compared with the OPCAB group (238.8 vs. 174.9 min; *p* < 0.001) and despite the requirement for complete left-lung deflation with prolonged single-lung ventilation (SLV), both surgical strategies were safe and effectively managed within a structured ERACS pathway, achieving comparable outcomes. Ventilation times, hospital LOS, unplanned ICU transfer rates, postoperative complications, and in-hospital mortality did not differ significantly between groups, indicating that the ERACS protocol effectively standardizes early recovery irrespective of surgical access.

Importantly, the comparison between MICS-CABG and OPCAB in the present study should not be interpreted as a comparison of surgical access alone. Rather, these approaches represent two distinct coronary revascularization strategies, each embedded within a broader procedural and perioperative framework that includes patient selection, anesthetic management, and anticipated recovery trajectory. At our institution, the choice of approach is driven by anatomical feasibility, physiological reserve, comorbidities, and technical considerations, resulting in two clinically appropriate but inherently different therapeutic populations. Within this context, our findings demonstrate that, when applied to appropriately selected patients and managed within a standardized ERACS pathway, both strategies can achieve comparable early postoperative recovery outcomes despite substantial differences in operative complexity, surgical exposure, and anesthetic demands—most notably the requirement for prolonged single-lung ventilation in MICS-CABG. This observation suggests that ERACS functions as an enabling perioperative framework capable of harmonizing early recovery across fundamentally different revascularization strategies, rather than merely optimizing outcomes for a specific surgical access technique.

MICS-CABG offers several potential advantages. Achieving complete revascularization while avoiding median sternotomy which reduces access trauma, eliminates the risk of sternal wound complications, and provides cosmetic and functional benefits that many patients prefer. The smaller incision may facilitate earlier mobilization and improve overall patient experience. These features align well with ERACS principles, which emphasize minimizing physiological stress, promoting early mobilization, and reducing postoperative opioid requirements.

However, these benefits must be balanced against the inherent limitations of the minimally invasive approach. MICS-CABG requires complete left-lung deflation and prolonged SLV, making adequate pulmonary function essential and excluding some patients. Limited exposure through a mini-thoracotomy increases technical complexity and contributes to longer operative times. Additionally, certain coronary anatomies—such as intramyocardial or heavily calcified targets—may limit suitability. A substantial learning curve and the need for specialized expertise further constrain widespread adoption.

Taken together, when applied to appropriately selected patients and performed by experienced teams, MICS-CABG reduces access-related surgical trauma without adversely affecting postoperative safety or efficacy. Importantly, our findings indicate that the longer operative times inherent to the minimally invasive approach can be accommodated within a standardized ERACS pathway, enabling reliable early extubation and ICU bypass. Overall, these results support the feasibility of both revascularization strategies within an ERACS-driven, PACU-based postoperative management framework.

Building on these findings, our results differ from several previous comparative studies that reported shorter postoperative ventilation times for MICS-CABG compared with OPCAB. Teman et al. [17] observed significantly shorter early ventilation for MICS-CABG despite longer operative times (MICS CABG 3.4 [1.8–5.5] h vs. OPCAB 4.9 [3.5–7.5] h; *p* < 0.001). In our cohort, however, both groups achieved exceptionally short ventilation times—approximately half of those previously reported—and no significant difference was observed between approaches (MICS-CABG 1.66 [1.2–2.2] h vs. OPCAB 1.75 [1.2–2.4] h; *p* = 0.59). These remarkably low values likely reflect institutional differences in postoperative management, particularly the structured ERACS-based PACU extubation pathway, which standardizes early postoperative care and minimizes variability.

With respect to hospital length of stay, earlier studies consistently reported shorter LOS after MICS-CABG [3,18,19]. In our study, however, LOS was comparable between approaches (MICS CABG 8.7 d vs. OPCAB 8.5 d; *p* = 0.78) and notably longer than in many prior publications. These discrepancies are likely attributable to differences in healthcare system regulations and discharge workflows rather than to perioperative management. Importantly, within our ERACS model, the choice of surgical access did not materially influence LOS.

Comparisons with previous literature regarding postoperative ICU utilization are similarly nuanced. Earlier fast-track studies from our center reported substantially higher ICU transfer or fast-track failure rates, ranging from 8 to 14% [8,14,16,20,21]. In contrast, the unplanned ICU transfer rate in the present study was very low in both groups (MICS CABG 1 patient 0.9% vs. OPCAB 3 patients 2.6%; *p* = 0.37). This likely reflects differences in patient risk profiles as well as the increasing experience of our team in managing structured early extubation pathways.

Transfusion patterns in previous studies have often favored MICS-CABG, with several reporting lower transfusion rates or reduced blood loss [3,17,19]. In our cohort, transfusion requirements tended to be lower in the MICS-CABG group, although the difference did not reach statistical significance. This may be explained by the restrictive transfusion policy and predefined thresholds used at our institution, which minimize practice variability and limit the impact of surgical access on transfusion decisions.

From a health-economic perspective, minimally invasive coronary surgery is often perceived as being associated with higher upfront procedural costs due to specialized instruments, longer setup times, and the need for dedicated surgical expertise, which may limit its adoption in resource-constrained healthcare systems. However, multiple multi-institutional and case-matched analyses have reported that, in real-world practice, these higher intraoperative costs are frequently offset by lower postoperative resource utilization, including shorter hospital and ICU stays, reduced transfusion requirements, and fewer wound-related complications, resulting in overall episode-of-care costs that have been reported as comparable to conventional off-pump CABG, particularly in high-volume centers with established expertise [17,22,23]. Although a formal cost analysis was beyond the scope of the present study, our findings support the concept that standardized ERACS pathways may help optimize resource utilization and maximize the value of minimally invasive strategies when applied in appropriately selected settings. This study has several limitations. First, its retrospective, single-center design introduces the potential for unmeasured confounding, and although propensity score matching was used to reduce baseline imbalances arising from intentional patient selection, residual confounding cannot be excluded. Second, the study was conducted in a high-volume center with extensive experience in both minimally invasive coronary surgery and PACU-based early extubation within an ERACS pathway; therefore, the findings may not be generalizable to centers using conventional postoperative ICU workflows or with different levels of institutional expertise. Third, postoperative cardiological and physiotherapeutic rehabilitation was not evaluated as a comparative outcome, as structured rehabilitation is routinely offered to all patients after major cardiac surgery within our healthcare system and allocation is largely determined by organizational availability rather than early postoperative clinical status or surgical approach. Finally, longer-term outcomes, including graft patency and late functional recovery, were beyond the scope of the present analysis and warrant evaluation in future prospective, multicenter studies.

## 5. Conclusions

In conclusion, our findings underscore that a well-implemented early-extubation ERACS protocol serves as a strong enabling framework for the minimally invasive era in cardiac surgery. When surgical safety and efficacy are ensured, ERACS protocol neutralizes other unique challenges inherent to each revascularization strategy, allowing both MICS-CABG and OPCAB to achieve comparable postoperative outcomes. Future multicenter studies are needed to confirm generalizability

## Figures and Tables

**Table 1:** Extubation Criteria: [16].

Parameter	Extubation Criteria
Consciousness	Fully awake and alert, no neurological deficit
Hemodynamic Stability	Stable without inotropic support
Core Temperature	≥36 °C
Arterial Blood Gases	PaO_2_ ≥ 100 mmHg, PaCO_2_ ≤ 42 mmHg on FiO_2_ 0.40
CentralVenous Oxygen Saturation (ScvO_2_)	>65%
Ventilation	Adequate tidal volumes with pressure support of 8 cmH_2_O and PEEP of 5 cmH_2_O
Chest drain output	<100 mL/h
Serum lactate	Within normal range
ECG and Chest X-ray	No new ischemic changes or pulmonary complications

**Table 2:** Transfer criteria from PACU to IMC: [16].

Parameter	Transfer Criteria
Consciousness	Fully awake, no neurological deficit
Hemodynamic Stability	Stable without significant support
Inotropic Support	None or minimal
Arterial blood gases	PaO_2_ > 90 mmHg, PaCO_2_ < 46 mmHg
Oxygen Saturation (SpO_2_)	>96% with 2–6 L/min O_2_
Urinary output	>0.5 mL/kg/h
Chest drain output	<50 mL/h, serous
Serum Lactate	Normal
Central Venous Oxygen Saturation (ScvO_2_)	>60%
Cardiac Enzymes & Chest X-ray	No findings requiring intervention
Pain Control	NRS < 4

**Table 3:** Demographic and intraoperative data. Values are presented as mean ± SD, median [IQR], and counts (%).

	MICS-CABG (n = 116)	OPCAB (n = 116)	*p*-Value
Age, years	64.72 ± 8.93	64.69 ± 9.56	0.983
Female sex	22 (19.0%)	18 (15.5%)	0.602
Height, cm	171.79 ± 8.24	174.01 ± 8.29	0.042
Weight, kg	81.31 ± 15.84	82.78 ± 13.53	0.449
BMI, kg/m^2^	27.48 ± 4.63	27.32 ± 4.12	0.788
LVEF, %	55.34 ± 11.04	55.34 ± 7.99	0.994
History of MI	40 (34.5%)	41 (35.3%)	0.999
EuroScore II	1.66 ± 1.67	1.53 ± 1.15	0.477
ASA II	3 (2.6%)	13 (11.2%)	0.017
ASA III	106 (91.4%)	98 (84.5%)	0.158
ASA IV	7 (6.0%)	5 (4.3%)	0.766
NYHA I	17 (14.7%)	14 (12.1%)	0.699
NYHA II	51 (44.0%)	66 (56.9%)	0.066
NYHA III	48 (41.4%)	35 (30.2%)	0.100
NYHA IV	0 (0.0%)	1 (0.9%)	0.999
Hypertension	97 (83.6%)	103 (88.8%)	0.341
Peripheral vascular disease	14 (12.1%)	5 (4.3%)	0.055
COPD	5 (4.3%)	6 (5.2%)	0.999
CNS disease	9 (7.8%)	9 (7.8%)	0.999
Chronic kidney disease	9 (7.8%)	13 (11.2%)	0.501
Diabetes mellitus	44 (37.9%)	35 (30.2%)	0.267
Smoker	28 (24.1%)	27 (23.3%)	0.999
Urgency	20 (17.2%)	17 (14.7%)	0.719
Redo surgery	3 (2.6%)	1 (0.9%)	0.621
Preoperative arrhythmia	10 (8.6%)	9 (7.8%)	0.999
Operative duration, min	238.88 ± 65.07; 225 [185–280]	174.96 ± 48.20; 165 [139–200]	<0.001
Number of grafts	2.31 ± 0.64; 2 [2–3]	2.33 ± 0.49; 2 [2–3]	0.817

Abbreviations: BMI, body mass index; LVEF, left ventricular ejection fraction; MI, myocardial infarction; ASA, American Society of Anesthesiologists physical status; NYHA, New York Heart Association class; COPD, chronic obstructive pulmonary disease; CNS, central nervous system.

**Table 4:** Comparison of primary endpoints. Values are presented as mean ± SD, median [IQR], and counts (%).

	MICS-CABG (n = 116)	OPCAB (n = 116)	Effect Size (MD/OR)	95% CI	*p*-Value
Ventilation time, min	112.20 ± 56.91; 100 [75–136]	116.87 ± 64.74; 105 [75–145]	4.67	−12.42 to 21.75	0.590
PACU LOS, min	262.08 ± 75.69; 258 [214–318]	265.04 ± 87.24; 250 [210–303]	2.96	−19.91 to 25.83	0.798
Unplanned ICU transfer	1 (0.9%)	3 (2.6%)	3.08	0.32 to 30.06	0.369
Hospital LOS, days	8.70 ± 3.97; 7 [7–9]	8.55 ± 4.09; 7 [6–9]	−0.15	−1.19 to 0.90	0.782

Abbreviations: PACU, post-anesthesia care unit; LOS, length of stay.

**Table 5:** Postoperative complications. Values are presented as mean ± SD, median [IQR], and counts (%).

	MICS-CABG (n = 116)	OPCAB (n = 116)	Effect (MD/OR)	95% CI	*p*-Value
Pericardial effusion	0 (0.0%)	1 (0.9%)	3.03	0.12 to 75.05	0.999
Myocardial infarction	18 (15.5%)	13 (11.2%)	0.69	0.32 to 1.48	0.440
Arrhythmia	38 (32.8%)	35 (30.2%)	0.89	0.51 to 1.54	0.777
Delirium	6 (5.2%)	9 (7.8%)	1.54	0.53 to 4.48	0.593
Stroke	3 (2.6%)	0 (0.0%)	0.14	0.01 to 2.72	0.246
Renal complications	8 (6.9%)	15 (12.9%)	2.00	0.82 to 4.93	0.187
Pulmonary complications	17 (14.7%)	14 (12.1%)	0.80	0.37 to 1.71	0.699
Significant bleeding	35 (30.2%)	27 (23.3%)	0.70	0.39 to 1.26	0.299
RBC transfusion (patients)	17 (14.6%)	28 (24.1%)	0.54	0.28 to 1.05	0.096
RBC units, all patients	0.40 ± 1.43	0.68 ± 1.41	−0.27	−0.62 to 0.07	0.120
RBC units, transfused only	2.76 ± 2.81	2.82 ± 1.51	−0.71	−1.57 to 0.16	0.103
Re-exploration	4 (3.4%)	2 (1.7%)	0.49	0.09 to 2.74	0.683
PONV	5 (4.3%)	3 (2.6%)	0.59	0.14 to 2.53	0.721
NRS pain score	2.03 ± 0.95; 2 [2–2]	1.90 ± 1.04; 2 [1–2]	−0.12	−0.38 to 0.14	0.347
In-hospital mortality	2 (1.7%)	0 (0.0%)	0.20	0.01 to 4.14	0.497

## Data Availability

The datasets generated and analyzed during the current study are available from the corresponding author on reasonable request.

## Abbreviations

The following abbreviations are used in this manuscript:

MICS-CABG	Minimally invasive coronary artery bypass grafting
OPCAB	Off-Pump Coronary Artery Bypass
ERACS	Enhanced Recovery after Cardiac Surgery
CPB	Cardiopulmonary Bypass
SLV	Single Lung Ventilation
